# Cytological and morphological analysis of hybrids between *Brassicoraphanus*, and *Brassica napus* for introgression of clubroot resistant trait into *Brassica napus* L

**DOI:** 10.1371/journal.pone.0177470

**Published:** 2017-05-15

**Authors:** Zongxiang Zhan, Chinedu Charles Nwafor, Zhaoke Hou, Jianfang Gong, Bin Zhu, Yingfen Jiang, Yongming Zhou, Jiangsheng Wu, Zhongyun Piao, Yue Tong, Chao Liu, Chunyu Zhang

**Affiliations:** 1 National Research Center of Rapeseed Engineering and Technology and College of Plant Science and Technology, Huazhong Agricultural University, Wuhan, China; 2 College of Life Science, Guizhou Normal University, Guiyang, China; 3 Crop Research Institute, Anhui Academy of Agricultural Sciences, Hefei, China; 4 Department of Horticulture, Shenyang Agricultural University, Shenyang, China; 5 Yichang Academy of Agriculture Science, Yichang, China; Agriculture and Agri-Food Canada, CANADA

## Abstract

Interspecific hybridization is a powerful tool for improvement of crop species, it has the potential to broaden the genetic base and create new plant forms for breeding programs. Synthetic allopolyploid is a widely-used model for the study of genetic recombination and fixed heterosis in Brassica. In *Brassica napus* breeding, identification and introgression of new sources of clubroot resistance trait from wild or related species into it by hybridization is a long-term crop management strategy for clubroot disease. Radish (*Raphanus sativus* L.) is a close relative of the Brassica and most radish accessions are immune to the clubroot disease. A synthesized allotetraploid *Brassicoraphanus* (RRCC, 2n = 36) between *R*. *sativus* cv. HQ-04 (2n = 18, RR) and *Brassica oleracea* var. alboglabra (L.H Bailey) (2n = 18, CC) proved resistant of multiple clubroot disease pathogen *P*. *brassicae*. To predict the possibility to transfer the clubroot resistance trait from the RR subgenome of allotetraploid *Brassicoraphanus* (RRCC, 2n = 36) into *Brassica napus* (AACC, 2n = 38), we analyzed the frequency of chromosome pairings in the F_1_ hybrids produced from a cross between *B*. *napus* cv. HS5 and the allotetraploid, characterize the genomic composition of some backcrossed progeny (BC_1_) using GISH, BAC-FISH and AFLP techniques. The level of intergenomic pairing between A and R genomes in the F_1_ hybrid was high, allosyndetic bivalents formed in 73.53% PMCs indicative of significant level of homeologous recombination between two genomes and high probability of incorporating chromosomal segments/genes from R-genome into A/C-genomes. The BC_1_ plants inherited variant extra R chromosomes or fragments from allotetraploid as revealed by GISH and AFLP analysis. 13.51% BC_2_ individuals were resistant to clubroot disease, and several resistance lines had high pollen fertility, Overall, the genetic material presented in this work represents a potential new genetic resource for practical use in breeding *B*. *napus* clubroot resistant cultivars.

## Introduction

Interspecific hybridization is a powerful tool for improvement of crop species, it has the potential to broaden genetic base and create new plant forms for breeding programs [1]. The efficiency of interspecific hybridization and gene transfer depends on the level of genetic and structural relatedness between the genomes of cultivated species and its wild relative [2]. Nowadays, synthetic allopolyploids are widely used to exploit related species for valuable agronomic trait through interspecific hybridization [3, 4]. They are mainly utilized as genetic bridge materials for introgression of target genes/regions that are absent in natural genetic background of cultivated crop species [5, 6], e.g. the Ogu-INRA cytoplasmic male sterility (CMS) system [6, 7] and clubroot disease resistance of *Raphanobrassica* [8].

In Rapeseed (*Brassica napus*) breeding, identification and introgression of new, stable and durable clubroot resistant traits from wild or closely related species into it, either through interspecific or intraspecific hybridization has been an important research objective for the improvement of this oilseed crop [9]. Because clubroot disease, pose a significant threat to rapeseed producing countries like Canada, Europe and China. For example, in China, since it emergence, production losses have increased by 30% [10]. The causal agent for clubroot disease is a soil-borne plant pathogen *Plasmodiophora brassicae* Woronin *(P*. *brassicae*) and previous studies have shown it can survived in soil as resting spores for many years making it difficult to be managed successfully by cultural, chemical and biological practices [11]. Interestingly some members of Brassica species maintain a range of locus diversity for clubroot resistance trait [12–16]. Nevertheless, evidence of partial or complete breakdown of several clubroot resistance genes identified in these Brassica species have emerged [16], perhaps due to wide variation for pathogenicity [17,18]. Overall, host plant genetic resistance remains the most sustainable and feasible alternative to combat the clubroot disease pathogen P. *brassicae*. Which underscores the need to identify, evaluate and introgress additional sources of clubroot resistance into *B*. *napus*. Indeed, interspecific and introgressive hybridization hold such potential [19], and pyramiding of different sources of clubroot resistance genes into a single line might provide alternative durable resistance to a broad spectrum of *P*. *brassicae*.

Analysis of clubroot resistance in a radish (*Raphanus sativus*) mapping population, identified a *Crs1* locus accounting for the resistance variation and cultivars carrying this locus were immune to *P*. *brassicae* [20]. In addition, characterization of a synthetic amphidiploid *Brassicoraphanus* (RRCC, 2n = 36) between *R*. *sativus* cv. HQ-04 (2n = 18, RR) and *Brassica alboglabra* Bailey (2n = 18, CC) showed that after numerous selfing generations and selection for seed set, plants with high fertility rate were obtained [3]. These authors concluded that the advantages of amphidiploid *Brassicoraphanus* (RRCC) includes resistance to clubroot disease [8], beet cyst nematode, and higher crossability with Brassica species.

Interestingly, both radish and rapeseed belong to the *Brassicaceae* family [21]; consequently, transferring the R genome from the synthetic amphidiploid *Brassicoraphanus* into A/C genomes of *B*. *napus* by hybridization could provide a new source of clubroot resistance trait. So far, there are no reports of how hybridization between the R genome of *R*. *sativus* and A/C genome of *B*. *napus* or the subsequent backcrossing to an A/C genome as a recurrent parent may impact the frequency of homologous and homeologous pairing during meiosis especially in their progeny. The number of homologous regions shared between radish (RR) and rapeseed (AACC) genomes is unknown. Moreover, how these shared regions associate to influence intergenomic exchanges remain elusive. This information will be indispensable in predicting the probability of intergenic exchanges between R and A/C genomes and the potential stability of an R genome fragments in the newly created hybrid.

In this study, we exploited the availability of a synthetic amphidiploid *Brassicoraphanus*, previously described by Chen (2008) [3]. This amphidiploid has a clubroot disease resistance phenotype [8]. With the purpose of using interspecific hybridization and recurrent backcrossing approach, to transfer clubroot resistance from *Brassicoraphanus*, into *B*. *napus*, (1) we analyzed the frequency of inheritance of different original chromosomes in their resulting F_1_ hybrid. (2) We demonstrate the production of hybrid lines carrying R-genome fragments and track these chromosome fragments using molecular markers and cytogenetic techniques at the BC_1_ stage of backcrossing.

## Materials and methods

### Plant materials

**Fig 1:**
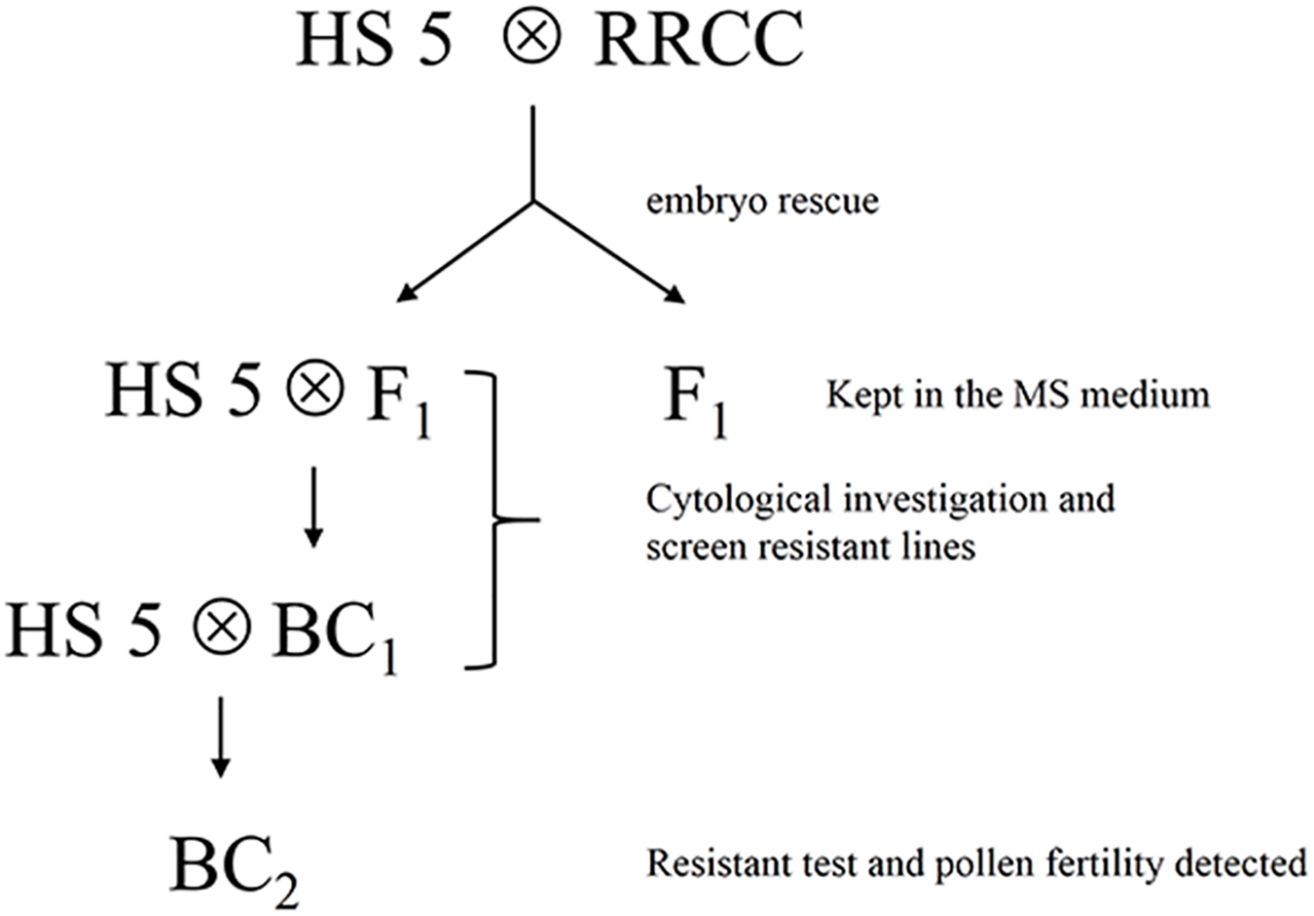
Flow chart of materials constructed.

Artificially synthesized amphidiploid *Brassicoraphanus* (RRCC, 2n = 36) from the intergenic hybridizations between *Brassica oleracea* L. (2n = 18, CC) and *Raphanus sativus* L. (2n = 18, RR) [3] was used as pollen parent in a cross with *B*. *napus* (2n = 38, AACC) cv. Huashuang 5 (HS5) to produce interspecific F_1_ hybrids (2n = 37, ARCC). The HS5 was the recurrent female parent in a subsequent backcross with F_1_ hybrids as male parent. The BC_2_ were from the resistant BC_1_ individual crossed with HS5. To obtain enough hybrid plantlets for this study, we produced four additional F_1_ hybrid plants from the initial cross through embryo rescue method [3]. All the plant materials generated from this work are described in (Fig 1).

### Test for clubroot resistance

Inoculation tests were carried out in the glasshouse according to the method described by Johnston (1968) [22]. Briefly, the frozen clubs were thawed at room temperature, ground and then mixed with dry peat soil in a ratio of 1:20. Inoculated soil was stored in dark condition for 48 h at 25°C. The seeds were sown in pots placed in a growth chamber at 25/20°C (day/night) with a photoperiod of 14 h at a light intensity of 200 mmol·m-2·s-1. The root symptoms evaluation of each plant was done 6 weeks after inoculation. The roots were washed carefully with tap water, visually examined for galls, and rated on a 0 to 3 scale [12], I.e. 0 = no visible gall formation or disease symptoms; 1 = a few small clubs, separate globular clubs on lateral roots; 2 = intermediate symptoms; 3 = severe clubs on main roots. Qualitative classification method was used to estimate clubroot resistance. I.e., a plant with no visible gall (zero score) was classified as resistant, while all other were recorded as susceptible. A total of 183 *Brassicoraphanus* plants and 171 HS5 individuals were used as resistant and susceptible controls to test four different pathogenic races of *P*. *brassicae*, i.e. race 2, 4, 7 and 10. Subsequently, 5 F_1_, 138 BC_1_, 84 BC_2_ and 98 HS5 lines were tested for resistance against *P*. *brassicae* race 4.

### Cytological investigation and pollen fertility analysis

Ovaries from young flower buds were harvested and treated with 2mM 8-hydroxyquinoline for 3 h. at room temperature, before fixing in Carnoy’s solution I (3:1 ethanol:glacial acetic acid, v/v). The fixed ovaries were stored at -20°C until use. For meiosis study, additional flower buds were fixed directly in Carnoy’s solution and stored at -20°C. Cytological observation was according to the procedure of [23]. Over 200 pollen grains from three flowers per plant of *Brassicoraphanus*, *B*. *napus*, F_1_ hybrid, BC_1_ and BC_2_ were stained with acetocarmine (1%), and the percentage of stained pollen grains analyzed in replicates to measure pollen fertility.

### DNA extraction, probe labeling and chromosome preparation

For total genomic DNA extraction, young leaves from Radish (*Raphanus sativus*) were gathered. Total genomic DNA extraction was according to the CTAB method. For genomic in situ hybridization (GISH) study, the Radish genomic DNA sample was labeled with digoxigenin-11-dUTP (Roche, Basel, Switzerland) using the BioPrime Array CGH Genomic Labeling System kit according to the manufacturer’s protocol (Invitrogen, Life Technologies). Next, a plasmid DNA of BAC BoB014O06 specific for C genome (provided by Z Li, Huazhong Agriculture University, Wuhan, China) was labeled with biotin-11-dCTP by the BioPrime DNA Labeling System kit (Invitrogen, Life Technologies). Total DNA of *B*. *rapa* (AA, 2n = 20) genotype extracted from *B*. *napus* [24] was prepared as blocking DNA by following the method of described by Cui (2012) [4]. Additionally, resynthesized plasmid DNA of BAC CL1 harboring 177bp satellite repeat sequences of *R*. *sativus* [25], was labeled with digoxigenin-11-dUTP and used as probe. Chromosome preparation protocol for FISH/GISH studies was as described by Cui (2012) [4].

### GISH and BAC-FISH analysis

GISH and FISH experiments was according to Cui (2012) [4] with minor modification. Briefly hybridization mixture for meiotic chromosomes consisted of 50% deionized formamide, 2× SSC, 10% dextran sulfate, 0.5% SDS, 100 ng radish probe, C-genome BAC-FISH probe, and 1000 ng block DNA per slide. For the analysis of somatic cells, hybridization mixture only contained C- and R- genome specific BAC-FISH probes. The mixture was denatured at 85°C for 10 min and cooled on ice for 5 min. Next, probes and chromosomes on slides were co-denatured for 7 min at 74°C in thermal cycler and hybridized at 37℃ overnight. The slides were washed stringently for 8 min in 0.1× SSC with 20% deionized formamide pretreated at 40°C. The immunodetection of biotinylated and digoxigenated probes were implemented with Cy3-labeled streptacidin (KPL, St.Louis) and anti-digoxigenin conjugate- Fluorescein isothiocyanate (FIFC) (Roch, Basel, Switzerland). Finally, the chromosomes on slides were counterstained with 4’-6-diamidino-2phenylindole (DAPI) solution followed by antifade solution (Vector Laboratories, Peterborough, UK). All images were captured with a CCD camera attached to a fluorescence microscope (Axio Scope A1, Zeiss, Germany). The chi-square contingency tests function of R software was used to determine the significant difference in pairing configuration.

### Sequence-related amplified polymorphism (SRAP) analysis

Genomic DNA of F_1_ hybrid was isolated using the CTAB technique. SRAP procedure was performed according to Li (2001) [26]. PCR Primer sequence were the same with those in the original protocol [26]. The amplification protocol was as follows: 5 cycles of 1min at 94°C, 1 min at 35°C, 1 min at 72°C, Annealing temperature was raised to 50°C for another 35 cycles [26]. The PCR products were visualized by silver staining and gel electrophoresis.

### Amplified fragment length polymorphism (AFLP) and clustering analysis

Genomic DNA was isolated from young leaves of *Brassicoraphanus*, *B*. *napus*, F_1_ hybrid and BC_1_ plants using the method of Doyle (1990) [27]. The DNA samples were prepared from each of the lines studied and were subjected to AFLP analysis. AFLP analysis was carried out using a modified protocol described by Vos (1995) [28]. In brief, the genomic DNA (250ng) was digested with *Eco*RI and *Mse*I for 1h at 37°C and then denatured for 20 min at 65°C. The adapters were ligated to the digested DNA with sticky end at 16°C overnight. The ligated products were used for PCR amplification; the reaction mixtures were diluted by 30-fold for selected amplification with 20 pairs of random selected primers. The PCR products were visualized by silver stain after 6% polyacrylamide gel electrophoresis. The DNA bands of BC_1_ generation and parents were analyzed with NTSYS Clustering software according to UPGMA [29].

## Results

### Morphology and cytology of hybrids (ARCC)

**Fig 2:**
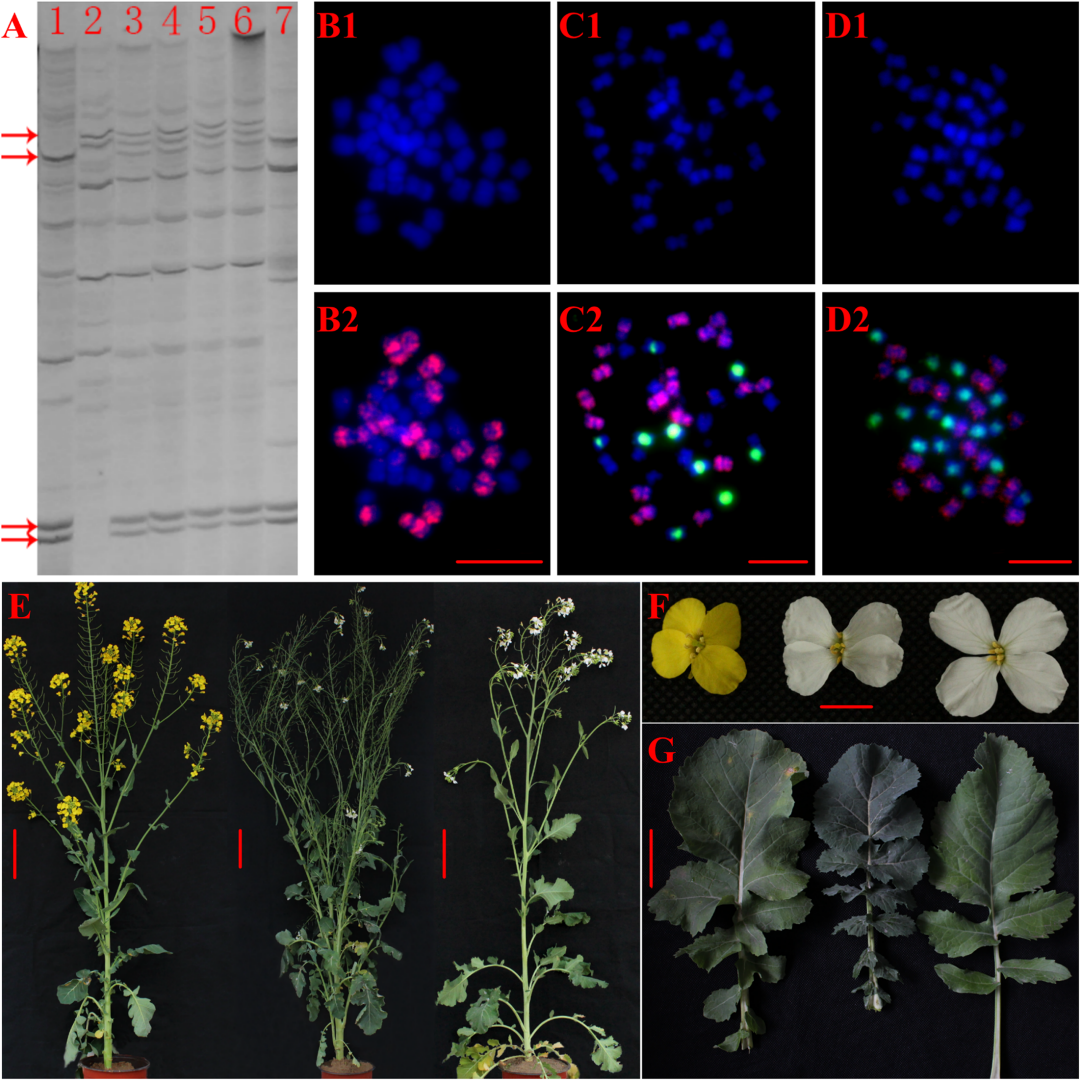
Morphology, cytological and SRAP profiles of hybrid and its parental lines. (A) Representative SRAP profiles generated from one primer pair in hybrids ARCC, parental lines and random selected radish variety, (line 1 = RRCC, line 2 = HS5, line 3–6 = the hybrids, line 7 = radish, red arrows: representative bands). (B-D) Somatic cell from HS5, F_1_ hybrid and RRCC. DAPI (blue) and merged images are given for each cell (left to right). Red signals are from C genome and green signals are from R genome in all the cells (bar: 10μm). (E) Parental lines and hybrid plant at full bloom (bar: 5cm). (F) Flowers (bar: 1cm), (G) Basal leaves (bar: 5cm).

To obtain the tri-genomic intergenic hybrid ARCC, HS5 (AACC, 2n = 38) was pollinated with pollens from *Brassicoraphanus* (RRCC, 2n = 36). All the progeny was confirmed hybrids by SRAP analysis and cytological observation (Fig 2A). Also, all the F_1_ hybrids had the expected chromosome number and complement (2n = 37, 10A+9R+18C) (Fig 2C). We observed obvious heterosis in vegetative growth among the F_1_ hybrids compared to their parents (Fig 2E). They exhibited intermediate flower morphology (Fig 2F). We noticed the hybrids had dark green leaves with similar serrated margins to HS5, but without long petioles observed in *Brassicoraphanus* (Fig 2J). Generally, they showed very low pollen fertility (4.86% viability).

### Variable chromosome pairing in F_1_ hybrid (ARCC)

**Fig 3:**
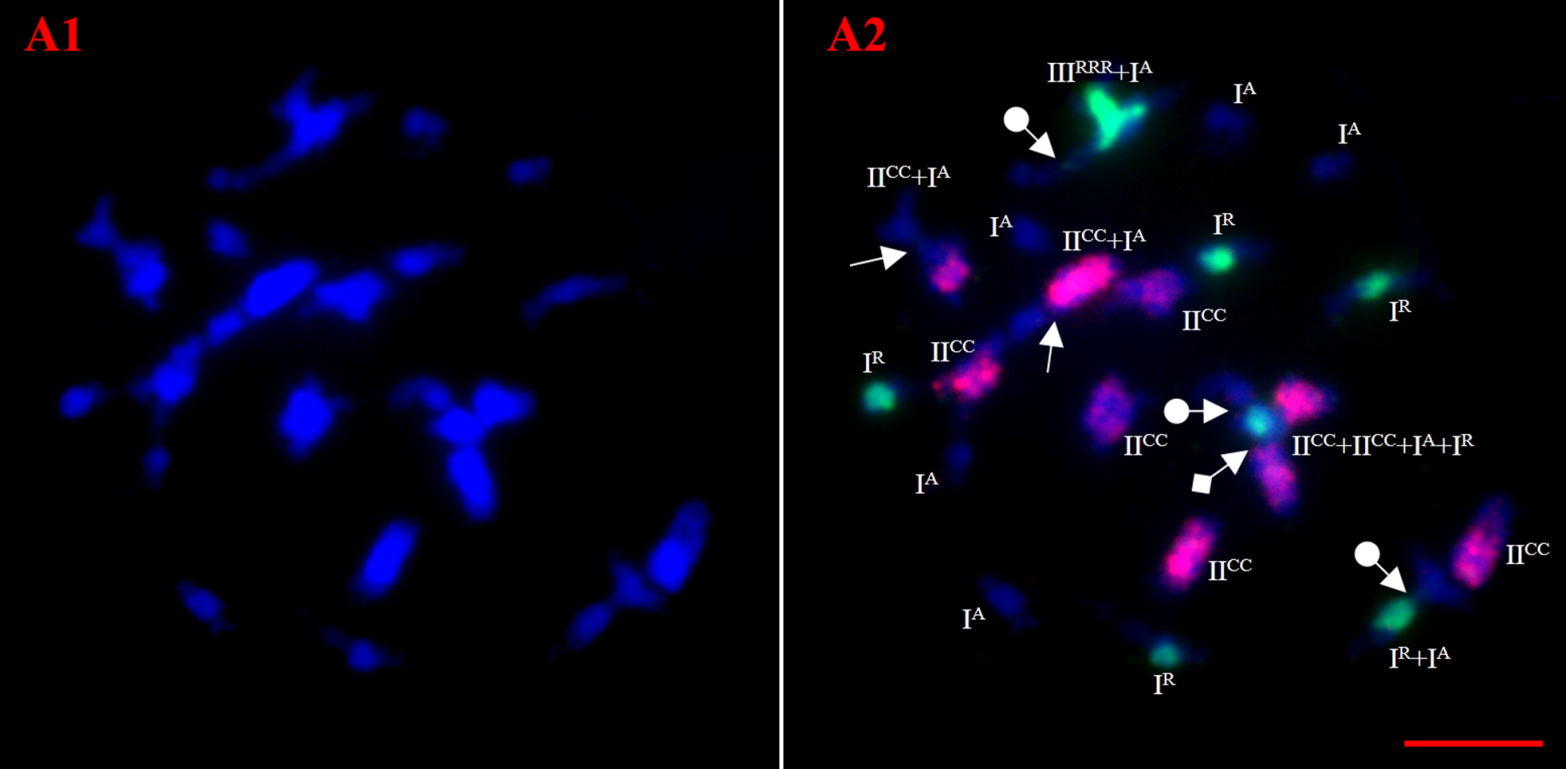
GISH-FISH analyses of meiotic chromosome pairings in PMCs of F_1_. (A1) DAPI (blue) and (A2) merged images of the representative cells at diakinesis. Red signals are from C genome; green signals are from R genome. (Solid arrow: C/A chromosomes pairing, solid arrow with ball tail: A/R chromosomes pairing, solid arrow with square tail: C/R chromosomes pairing, bar: 10μm).

Multi-color FISH readily distinguished the chromosomes of three genomes of the hybrids (ARCC) in pollen mother cells (PMCs) with two probes for C- and R genomes (Fig 3), which revealed the various intra-/intergenomic pairing. Our result showed the A and R-genomes were in a haploid state and C-genome was in diploid state, therefore unpaired chromosomes in PMCs at diakinesis and metaphase I (MI) were those from A and R genomes. The absence of univalents from C genome in PMC is because of the diploid state of the C-genome in the hybrid (Table 1) [29]. Furthermore, chromosome association at MI showed the A-genome and R-genome univalents appeared in 88.24% and (82.35%) PMC respectively. Similarly, the average univalent (2.70) for the A-genome was higher than that of R-genome (2.03), but not significantly different (χ2 = 1.52, p>0.05).

**Table 1:** Chromosome associations in PMCs of hybrids at diakinesis revealed by GISH.

	I			II				III					others		Pollen fertility (%)
	I^A^	I^R^	I^C^	II^AA^	II^RR^	II^AR^	II^CC^	III^ACC^	III^CCR^	III^RRR^	III^ARR^	III^AAR^		Total PMCs	mean ±dev.st
average	2.7	2.03	0	0.35^a^	0.68^b^	1.29^c^	4.71	2.15^a^	0.29^b^	0.15	0.32	0.09	1.94	34	4.86±2.06
range	0–7	0–5	-	0–2	0–2	0–4	2–8	0–4	0–2	0–2	0–2	0–1	0–4		
percentage	88.24%	82.35%	0	29.41%	47.06%	73.53%	100%	91.18%	20.59%	11.76%	26.47%	8.82%	-		

I, univalent, II, bivalent, III, trivalent. I^A^, I^R^, I^C^ indicate univalent belonging to the A, R, C genomes, respectively. II^AA^, II^RR^ indicate autosyndetic bivalent formed between chromosomes of A, R genomes. II^AR^ indicates allosyndetic bivalents formed between A and R chromosomes (Cui et al., 2012),

^a, b, c^ group significantly different by χ^2^ –test, p<0.05.

Besides the unpaired chromosomes, we observed both A and R genomes formed autosyndetic bivalents in 47.06% and 29.41% of PMCs respectively, although the autosyndetic bivalents from R genome was much higher than for A-genome. The maximum number of autosyndetic bivalents was 2 for both R-genome and A-genome respectively, but the average autosyndetic bivalents for R-genome (0.68) was significantly higher than that of A-genome (0.35), (χ2 = 4.248, p<0.05). The maximum number of autosyndetic trivalents of the R-genome was 2 and appeared in 11.76% PMCs, with an average of 0.15 (Table 1), while no such pairing was observed for A-genome. We detected autosyndesis within A and R genomes in the formation of allosyndetic trivalents A-R-R and A-A-R. The average of A-R-R (0.32) was much higher than A-A-R (0.09), the higher rate of autosyndesis within R-genome suggested the higher degree of homeology among the R-genome chromosomes.

As for allosyndesis or intergenomic pairing between A and R genomes, the allosyndetic bivalents formed in 73.53% PMCs, having the maximum and average of 4 and 1.29. The frequency of allosyndesis (1.29) was significantly higher than that of autosyndesis (0.35 for A-genome; 0.68 for R-genome, χ2 = 18.70, p<0.05; χ2 = 6.70, p<0.05), showing the higher level of homeology between two genomes than within each genome. Intergenomic pairings between A/C and R genomes also appeared by producing the allosyndetic trivalents, A-A-R,A-R-R, and C-C-R, in 8.82%, 26.47%, and 20.59% PMCs, respectively. The averages of the trivalents A-R-R (0.32) and C-C-R (0.29) were comparable but significantly higher than that for A-A-R (0.09). For C genome in diploidy state, the homologous pairings prevailed by forming bivalents in all PMCs, but the number of bivalents varied in wide ranges (2–8) and averaged only 4.71, much fewer than the theoretical value 9. Besides the allosyndetic trivalent C-C-R, the A-C-C type appeared in 91.18% PMCs, with the maximum 4 and the mean 2.15. Moreover, it was the highest frequency (2.15) of allosyndesis resulting from the close relationship between A and C genome. Notably, only two forms of allosyndesis (A-C-C, C-C-R) including two C-genome chromosomes and one other were observed, and no A-C or C-R bivalents or C-R-R trivalent were produced, possibly showing the priority of homologous pairing. Interestingly, the average of the trivalents A-R-R (0.32) was significantly higher than A-A-R (0.09) which likely resulted from the higher rate of R-R bivalents than A-A bivalents, or the A-genome chromosomes were involved in the formation of the C-C-A trivalents; the higher rate of C-C-R (0.29) than A-A-R were attributable to the more C-C bivalents than A-A ones.

### Morphological, cytological characterization and AFLP analysis in the BC_1_

**Fig 4:**
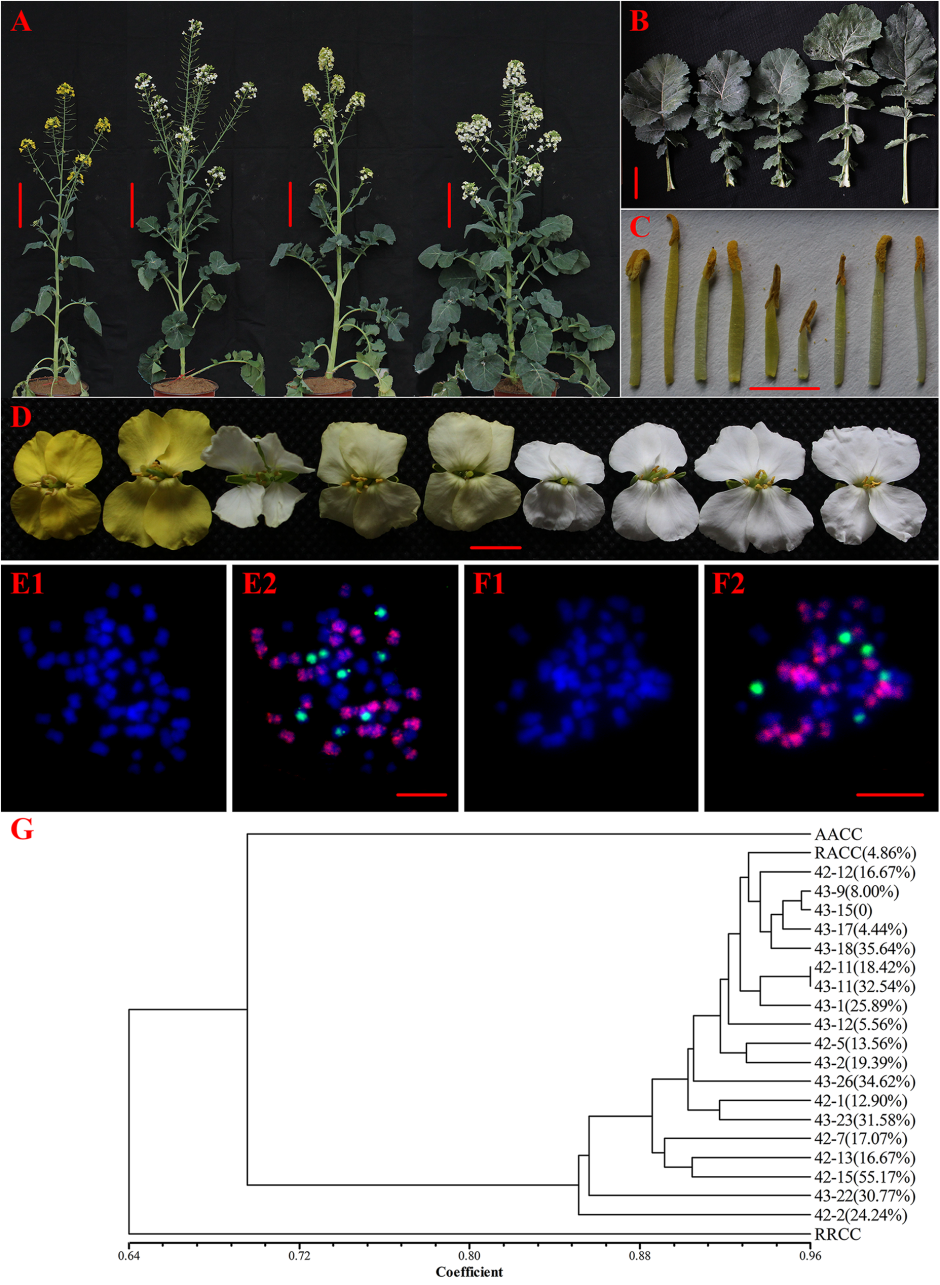
Morphology, cytological and AFLP analysis of some BC_1_ individuals and their parental lines. (A) BC_1_ plants at full bloom (bar: 15cm), (B) Flowers from HS5, seven different BC_1_ individuals and F_1_ (left to right), (C) Basal leaves, (D) Longer stamen from the flower of B (bar 0.5cm), (E-F) Somatic cell from two randomly selected BC_1_ individuals (bar: 10μm). DAPI (blue) and merged images are given for each cell (left to right). Red signals are from C genome green signals are from R genome in all the cells (bar: 10μm). (G) AFLP clustering analysis of two parental line, F_1_ hybrid and randomly selected BC_1_ plants.

In total 138 BC_1_ plants were analyzed for morphological traits. Fewer hybrids showed strong vigorous vegetative growth (Fig 4A). Majority of hybrids showed intermediate flower and leaf morphology (Fig 4B); nevertheless, few of the hybrids had leaves with similar long petiole to *Brassicoraphanus* (Fig 4C). Stamen development and pollen fertility varied among the hybrids, majority were relatively fertile although a few of the hybrids were male sterile and had stunted stamens (Fig 4D and S1 Table).

The cytological results indicated that the R chromosome was introgressed from F_1_ to BC_1_ generation (Fig 4E and 4F). The chromosome composition of randomly selected two individuals (No. 43–22 and 44–13) of BC_1_ showed a relatively regular and irregular pairing, i.e., chromosome constitution of No. 43–22 was 2n = 38, 15A+5R+18C, while that of No. 44–13 was 2n = 51, 18A+8R+25C. We observed that the number of chromosomes in No. 43–22 was less than that of No. 44–13. Similarly, comparison of pollen germination and viability test indicate No. 43–22 had significantly high pollen fertility (p<0.05) than No. 43–13, possibly due to irregular chromosome complement of 44–13.

To determine the contribution of parental genomes in the backcross progeny, we randomly selected 19 BC_1_ individuals for AFLP analysis. By using 18 AFLP primer combinations, we obtained 231 bands, of which 134 bands were polymorphic S2 Table. Similarity coefficient among BC_1_ generation ranged from 0.85 to 0.96, suggesting they have identical genetic composition (Fig 4G). Most BC_1_ individuals (No. 42–2, 43–22 and 42–15) resembled HS5 than *Brassicoraphanus*, and had relatively high pollen fertility. On the other hand, a few BC_1_ individuals (43–15, 43–39 and 43–17) that resemble F_1_ hybrid showed low (0–9%) pollen fertility.

### Clubroot resistant test of the parental lines F_1_ and BC_1_

**Fig 5:**
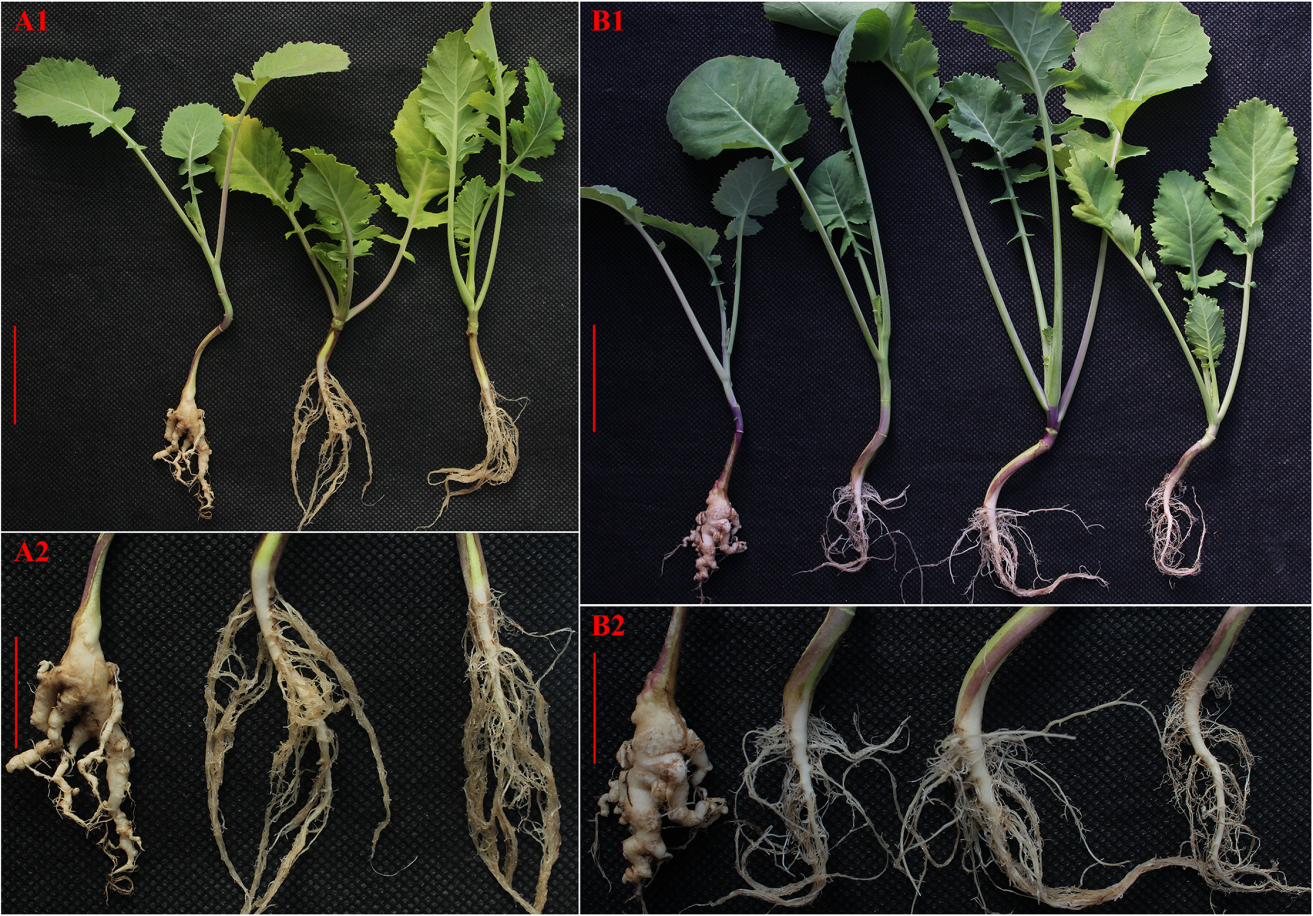
Clubroot resistant test with race 4. (A1) Root symptoms evaluation of HS5, hybrid and RRCC resistant characters (left to right, bar: 5cm), (A2) Enlarged image of A1 (bar: 2.5cm). (B1) Root symptoms evaluation of HS5, randomly selected BC_1_ individuals and RRCC (left to right bar: 5cm). (B2) Enlarged image of B1 (bar: 2.5cm).

We evaluated the parental lines ‘*Brassicoraphanus* (RRCC)’ and ‘HS5 (AACC)’, the F_1_ hybrids (ARCC) and BC_1_ generation for clubroot resistance. The parental lines were inoculated with four (4) pathogenic races of *P*. *brassicae* that are wide spread in China. Namely, *P*. *brassicae* race No. 2, 4, 7 and 10. The root symptom evaluation of parent lines revealed that all *Brassicoraphanus* lines, had no symptoms of infection or gall development when tested with the 4 pathogenic races, consequently they were categorized as resistant (See methods and Table 2). Conversely, the HS5 lines showed obvious signs of very high susceptibility to these pathogens (Table 2). Due to difficulty in obtaining seeds after performing numerous crossing between the parental lines, F_1_ and BC_1_ resistance test was performed with only pathotype race No. 4 of *P*. *brassicae* isolates since it is the most virulent pathogenic form of *P*. *brassicae* dominant in China [30]. All the F_1_ plants challenged with *P*. *brassicae* race No. 4, showed the same resistant phenotype as the parental line *“Brassicoraphanus* (RRCC)” (Table 3). However, root symptom evaluation revealed that BC_1_ plants showed segregation phenotype to *P*. *brassicae* resistance i.e., majority (106 out of 138 plants) of the BC_1_ individuals showed no sign of infection or gall development like the F_1_ generation and were classified resistant (Table 3). On the contrary, twenty-six (26) BC_1_ plants had some disease symptoms i.e., small but visible galls on lateral roots, while the remaining six (6) BC_1_ plants showed high disease symptoms severity with severe clubs on main roots (Table 3 and Fig 5B), consequently we classified both lines susceptible (Table 3). Taken together our result demonstrate that some of the BC_1_ individuals are carrying R-genome fragments or region containing the clubroot resistant allele.

**Table 2:** Qualitative classification of clubroot resistance of parental lines tested with different pathogenic race.

Pathogenic race	2		4		7		10	
Plant materials	RRCC	HS5	RRCC	HS5	RRCC	HS5	RRCC	HS5
**Resistant**	46	0	48	0	52	0	37	0
**Susceptible**	0	52	0	42	0	45	0	32

**Table 3:** Qualitative resistance of hybrids and BC_1_ generation tested with race No. 4 of *P*. *brassicae*.

	High Susceptibility	Low susceptibility	Resistant
**HS5**	42	0	0
**RRCC**	0	0	48
**ARCC**	0	0	5
**BC**_**1**_	6	26	106

### Clubroot resistant test of the BC_2_ and pollen fertility of the resistant lines

**Fig 6:**
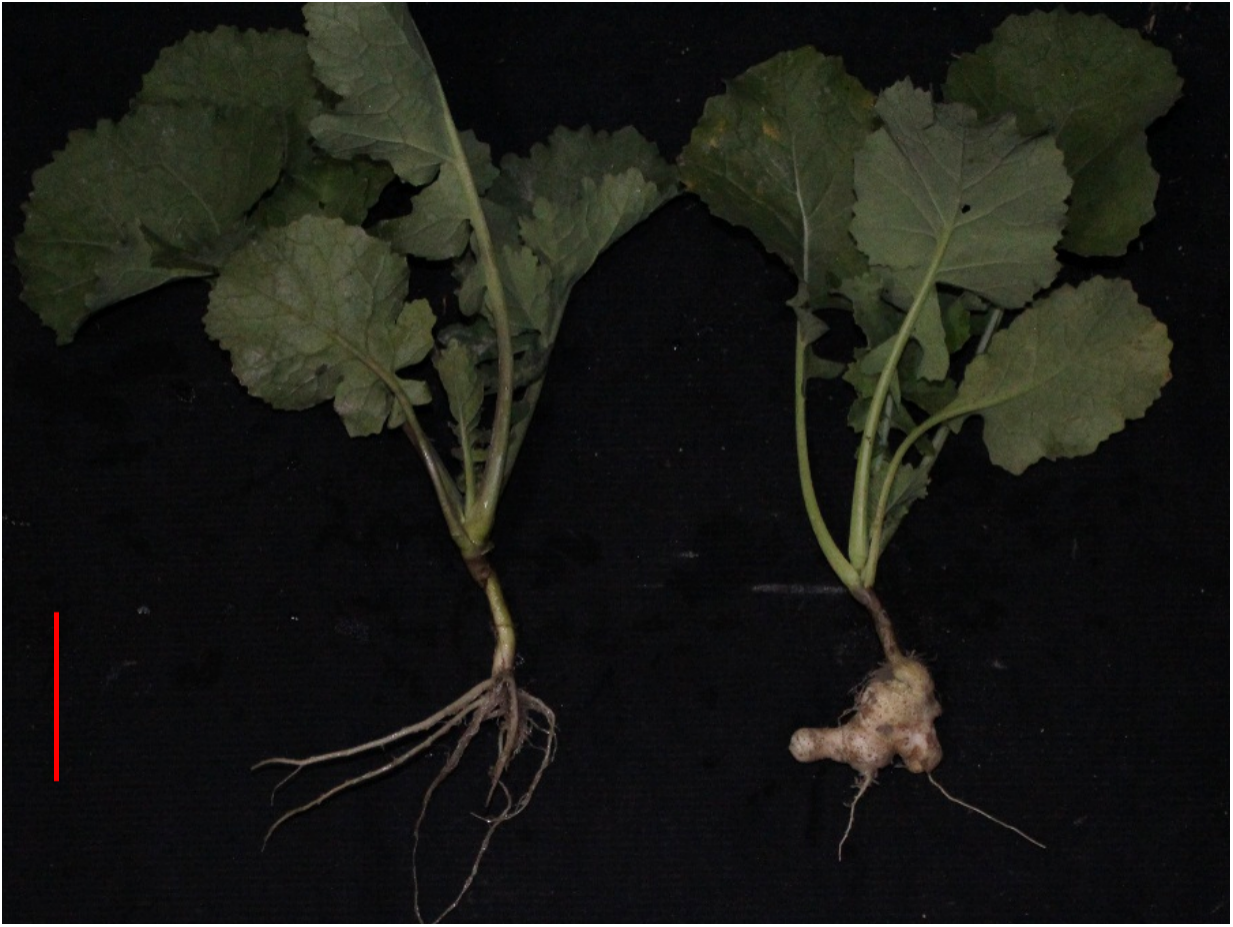
Clubroot resistant test in disease field. Root symptoms evaluation of representatively selected resistant BC_2_ individuals and control plants HS 5 (left to right, bar: 5cM).

We crossed the resistant BC_1_ lines with HS5, and obtained 74 BC_2_ population. To select the resistant lines from BC_2_ generation, all BC_2_ lines and control line HS5 were planted in the field severely infested with clubroot disease pathgentype of No. 4. The infection profile of the clubroot disease pathgentype of No. 4 indicate that 13.51% of 74 BC_2_ individuals were immune to the disease affection, which was significantly higher than that of control materials (3.06% of 98 plants, Fig 6), Also pollen fertility improved greatly in these progenies compared with BC_1_ ranging from 37.07%- 98.64%. Obviously, most fertile BC_2_ individuals were like control line HS5 (S3 Table and S1 Fig). However very few resistant BC_2_ individuals (lines 17–2, 17–3, 17–7, 17–9) had low pollen fertility, while the rest had excellent pollen fertility which was still significantly higher than that of control (S3 Table).

## Discussion

Synthesized allopolyploids are important hybridization tool for chromosome manipulation targeted at crop improvement [31]. In this study, we try to transfer clubroot resistance trait in the R-genome of synthesized *Brassicoraphanus* (RRCC, 2n = 36) [3], to *B*. *napus* cv. HS5 (AACC, 2n = 38). This resistance originally was derived from *Raphanus sativus* clubroot resistant type, and previously used by Mcnaughton (1973) [8] to synthesize intergeneric amphidiploids *Brassicoraphanus* and recently by Chen (2008) [3] to produce F_10_ plants with good fertility after several backcrossing. Our data demonstrate that we have successfully produced F_1_ hybrids (ARCC. 2n = 37) and BC_1_ progenies that contain R-genome segments at the same time resistant to clubroot disease pathogene *P*. *brassicae* (Table 3). To facilitate genetic characterization of F_1_ hybrid and the new clubroot resistance trait, we analyzed the frequency of chromosome pairings in the F_1_ hybrids and genomic composition of some BC_1_ progenies by GISH and BAC-FISH [32]. additionally, shed light on the impacts of chromosome recombination rates of auto- and allosyndesis on the hybrid genomic structure, stability of the genomes at the ploidy levels and hybrid morphology. Equally, the combination of molecular marker analysis and pollen fertility assays provided a rare opportunity to study the inheritance of the R-genome/chromosomes and clubroot resistance trait in the BC_1_ progenies, underscoring the usefulness of synthetic allopolyploids Brassica [3].

### Recombination frequency of the R-genome in the F_1_ hybrid

Homeologous exchanges have been shown to drive introgressive hybridization in Gossypium [33], and increase genetic variation observed for important agronomic traits like Fusarium head blight resistance in durum wheat [34], beet cyst nematode resistance [35], and cytoplasmic male sterility in Brassica [36]. In the present study, we analyzed the extent of homeologous exchanges between the R and A genomes, in an interspecific F_1_ hybrid (ARCC) produced from a cross between *Brassicoraphanus* (RRCC, 2n = 36) and *B*. *napus* (AACC, 2n = 38). Our result showed high homeologous recombination rate (73.53% of PMCs) between the R and A genomes of the F_1_ hybrid at diakinesis (Table 1). Similarly, half the number of R chromosome was inherited in the F_1_ hybrid (ARCC, 2n = 37, 10A+9R+18C), which implied a high potential of transferring clubroot resistant gene from R genome into A or C genome of rapeseed. Homeology between the genomes of Radish (RR) and Brassica species has been widely reported, and used to transfer useful traits from Radish to Brassica species through both conventional and somatic hybridization techniques [37]. For instance, Singh and co-workers reported that high frequent homeologous pairing was responsible for the genome affinity observed between A/C and Rr genomes in an intergeneric F_1_ hybrid between *B*. *napus* (AC, n = 19) and *Raphanus raphanistrum* (Rr, n = 9) [38]. Also, researchers have reported the transfer of a nuclear-encoded fertility restorer (Rf) gene from *R*. *sativus* (RR) to *B*. *napus* (AACC) by synthesis of RRAACC hybrid by sexual crosses [7, 37]. Equally, Arumugam and co-workers fused protoplasts of RC hybrid obtained through sexual crosses between *R*. *sativus* and *B*. *oleracea* (male) with the protoplast of *B*. *nigra* (BB) to produce three genome-hybrids RCBB with the aim of introgressing heat tolerance in Brassica species [39]. Taken together our results and other studies confirmed that the transfer of clubroot resistant alleles/genes from *R*. *sativus* to *B*. *napus* through homeologous recombination between A, C and R genomes is eminently possible. Several cytological studies have shown high frequency of homologous chromosome pairing among C subgenomes of Brassica species [14, 30, 38]. Our results showed that the C subgenomes in *Brassicoraphanus* (RRCC, 2n = 36) and *B*. *napus* (AACC, 2n = 38) were in a disomic stage therefore it is not surprising that regular homologous pairing occurred almost 100% in the PMCs in their F_1_ hybrid.

Considering amount of trivalents formed in the F_1_ hybrid PMCs, our results showed the R chromosomes could relatively pair with a bivalent C-genome (C-C-R, 0.29) and simultaneously with A-genome in trivalent combinations (A-R-R, 0.32., and A-A-R, 0.09), suggesting a possible suppression of the *PrBn* (Pairing regulator in *B*. *napus*) locus [40]. It might be worthy of mention that these results further validate the INRA-ogura CMS systems were *Rfo* genes have been successfully transferred from radish into the C-genome of rapeseed [41–43]. In addition, we noticed the chromosomes of A-genome paired easily with chromosomes of diploid C-genome (C-C-A, 2.15), which is consistent with previous studies [44]. Finally, we have provided the chromosomal evidence that the R-genome could be transferred to the A/C genome of *B*. *napus* (Table 1) which could be useful in *B*. *rapa*, *B*. *carinata* and *B*. *juncea* breeding as demonstrated in other crops [45,46].

### Morphology and pollen fertility of the F_1_ and BC_1_

Homeologous recombination can generate hybrid plants with novel genomic compositions and useful phenotypes [47]. In the present study, we confirmed all F_1_ plants to be hybrids with intermediate morphology. Similarly, most of the morphological attributes of BC_1_ progenies were intermediate of their parents. Although, a few of the BC_1_ plants had leaves petiole like *Brassicoraphanus* (Fig 4B). Several studies have shown that reproductive barriers exist in the intergenic or interspecific F_1_ hybrids of Amphidiploids, probably due to, limited chromosome homology, low rates of recombination, pollen abnormality, sterility or low fertility and hybrid incompatibility [3, 48, 49]. In the present study, we found pollen fertility of the F_1_ hybrids highly abnormal (Table 1), even with a balanced chromosome composition of ARCC. Perhaps this could explain the very low number of seeds (F_1_) obtained after the initial crossing between the parental lines. Although the pollen fertility levels slightly increased in the BC_1_ especially as progenies recover to HS5 (Fig 4G), it did not eliminate the fertility problem (fewer seeds) because the highest fertility rate among the BC_1_ individuals was about 21% (S1 Table) which was considered low. In the future, we hope to overcome this barrier by performing repeated backcrossing to HS5.

### Clubroot disease resistance

Based on the result of qualitative classification, all *Brassicoraphanus* lines and F_1_ hybrids were resistant to clubroot disease (Tables 2 and 3). It follows that, qualitative classification revealed about 77% of the BC_1_ and 13.51% of BC_2_ progenies were resistant to clubroot disease pathotypes race 4 (Table 3), and the pollen fertility of resistant BC_2_ plants improved greatly, some of which were similar to the control plants. For the reason, firstly, as reported by Japanese group that clubroot resistance character is controlled by single dominant gene localized in *Csr1* locus [20]; and secondly, in BC_1_ generations, the plants still have variant extra R chromosomes from radish as revealed by GISH and confirmed by AFLP. Thirdly the chromosomes in BC_1_ progeny were very unstable, which resulted in the disappearance of R chromosome in BC_2_. Therefore, it is tempting to speculate that the high frequency of resistance observed in the BC_1_ generation may due to a single dominant locus Although these results might be considered preliminary, however, we have obtained reliable resistant BC_2_ lines with normal pollen fertility. Therefore, future work will focus on BC_2_ lines with high pollen fertility and stable chromosome composition, carrying clubroot disease resistant locus, which is more suitable for QTL mapping.

## Conclusion

In summary, we have successfully produced F_1_ hybrid (ARCC. 2n = 37), BC_1_ and BC_2_ progenies that show higher resistance to clubroot disease pathogenic *P*. *brassicae*. The current material allowed us, for the first time to demonstrate the translocation and introgression of R genome from radish into rapeseed A/C genome background at a higher frequency of chromosome recombination. The clubroot resistant material generated in this work represents a potential new genetic resource not only for combating *P*. *brassicae*, also other useful characters can be exploited for Brassica breeding.

## Supporting information

S1 Table Pollen fertility in randomly selected BC_1_ individuals. Abnormal pollen fertility of F_1_ hybrids and BC_1_ individuals.
(DOCX)

S2 Table Summary of AFLP bands in random selected BC_1_ individuals. AFLP analysis identified polymorphic bands among randomly selected 19 BC_1_ individuals.
(DOCX)

S3 Table Pollen fertility of backcross parent and resistant BC_2_ individuals. (DOCX)

S1 Fig **Pollen fertility of representatively individuals (A) excellent pollen (B) low pollen**.
(TIF)

## References

[1] Chandra A, Gupta ML, Ahuja I, Kaur G, Banga SS. Intergeneric hybridization between *Erucastrum cardaminoides* and two diploid crop *Brassica* species. Theoretical and Applied Genetics. 2004;108(8):1620–6. doi: 10.1007/s00122-004-1592-1 14985971 10.1007/s00122-004-1592-1

[2] Leflon M, Eber F, Letanneur JC, Chelysheva L, Coriton O, Huteau V, et al Pairing and recombination at meiosis of *Brassica rapa* (AA) x *Brassica napus* (AACC) hybrids. Theoretical and Applied Genetics. 2006;113(8):1467–80. doi: 10.1007/s00122-006-0393-0 16983552 10.1007/s00122-006-0393-0

[3] Chen HG, Wu JS. Characterization of fertile amphidiploid between *Raphanus sativus* and *Brassica alboglabra* and the crossability with Brassica species. Genetic Resources and Crop Evolution. 2008;55(1):143–50.

[4] Cui C, Ge X, Gautam M, Kang L, Li Z. Cytoplasmic and genomic effects on meiotic pairing in Brassica hybrids and allotetraploids from pair crosses of three cultivated diploids. Genetics. 2012;191(3):725–38. doi: 10.1534/genetics.112.140780 22505621 10.1534/genetics.112.140780 PMC3389969

[5] Grandont L, Coriton O, Huteau V, Eber F, Grelon M, Chelysheva L, et al Homoeologous chromosome sorting and progression of meiotic recombination in *Brassica napus*: Ploidy Does Matter! 2014;26(4):1448 doi: 10.1105/tpc.114.122788 24737673 10.1105/tpc.114.122788 PMC4036564

[6] Yamagishi H, Bhat SR. Cytoplasmic male sterility in Brassicaceae crops. Breeding Science. 2014;64(1):38 doi: 10.1270/jsbbs.64.38 24987289 10.1270/jsbbs.64.38 PMC4031109

[7] Paulmann M, Röbbelen G. Effective transfer of cytoplasmic male sterility from Radish (*Raphanus sativus* L.) to Rape (*Brassiest napus* L.). Plant Breeding. 1988;100(4):299–309.

[8] Mcnaughton IH. Resistance of *Raphanobrassica* to clubroot disease. Nature. 1973;243(5409):547–8.

[9] Ky CL, Barre P, Lorieux M, Trouslot P, Akaffou S, Louarn J, et al Interspecific genetic linkage map, segregation distortion and genetic conversion in coffee (*Coffea* sp.). Theoretical and Applied Genetics. 2000;101(4):669–76.

[10] Chai AL, Xie XW, Shi YX, Li BJ. Research status of clubroot (*Plasmodiophora brassicae*) on cruciferous crops in China. Canadian Journal of Plant Pathology. 2014;36(sup1):142–53.

[11] Voorrips RE. *Plasmodiophora brassicae*: aspects of pathogenesis and resistance in *Brassica oleracea*. Euphytica. 1995;83(2):139–46.

[12] Suwabe K, Tsukazaki H, Iketani H, Hatakeyama K, Fujimura M, Nunome T, et al Identification of two loci for resistance to clubroot (*Plasmodiophora brassicae* Woronin) in *Brassica rapa* L. Theoretical and Applied Genetics. 2003;107(6):997–1002. doi: 10.1007/s00122-003-1309-x 12955203 10.1007/s00122-003-1309-x

[13] Sakamoto K, Saito A, Hayashida N, Taguchi G, Matsumoto E. Mapping of isolate-specific QTLs for clubroot resistance in Chinese cabbage (*Brassica rapa* L. ssp. pekinensis). Theoretical and Applied Genetics. 2008;117(5):759–67. doi: 10.1007/s00122-008-0817-0 18612625 10.1007/s00122-008-0817-0

[14] Navabi ZK, Stead KE, Pires JC, Xiong Z, Sharpe AG, Parkin IA, et al Analysis of B-genome chromosome introgression in interspecific hybrids of *Brassica napus* × *B*. *carinata*. Genetics. 2011;187(3):659 doi: 10.1534/genetics.110.124925 21196520 10.1534/genetics.110.124925 PMC3063663

[15] Rahman H, Shakir A, Hasan MJ. Breeding for clubroot resistant spring canola (*Brassica napus* L.) for the Canadian prairies: Can the European winter canola cv. Mendel be used as a source of resistance? Canadian Journal of Plant Science. 2017;91(3):447–58.

[16] Kato T, Hatakeyama K, Fukino N, Matsumoto S. Identification of a clubroot resistance locus conferring resistance to a *Plasmodiophora brassicae* classified into pathotype group 3 in Chinese cabbage (*Brassica rapa* L.). Breeding Science. 2012;62(62):282–7. 23226089 10.1270/jsbbs.62.282 PMC3501946

[17] Hirai M. Genetic analysis of clubroot resistance in Brassica crops. Breeding Science. 2006;56(3):223–9.

[18] Diederichsen E, Frauen M. Status and perspectives of clubroot resistance breeding in crucifer crops. Journal of Plant Growth Regulation. 2009;28(3):265–81.

[19] Guttman B. Evolution. Encyclopedia of Genetics. 2001;49(10):663–6.

[20] Kamei A, Tsuro M, Kubo N, Hayashi T, Wang N, Fujimura T, et al QTL mapping of clubroot resistance in radish (*Raphanus sativus* L.). Theoretical and Applied Genetics. 2010;120(5):1021 doi: 10.1007/s00122-009-1230-z 20012934 10.1007/s00122-009-1230-z

[21] Kaneko Y, Yano H, Bang SW, Matsuzawa Y. Production and characterization of *Raphanus sativus*-*Brassica rapa* monosomic chromosome addition lines. Plant Breeding. 2001;120(120):163–8.

[22] Johnston TD. Club root in Brassica: a standard inoculation technique and the specification of races. Plant Pathology. 2007;17(4):184–7.

[23] Li Z, Liu HL, Luo P. Production and cytogenetics of intergeneric hybrids between *Brassica napus* and *Orychophragmus violaceus*. Theoretical and Applied Genetics. 1995;91(1):131–6. doi: 10.1007/BF00220869 24169678 10.1007/BF00220869

[24] Tu Y, Sun J, Liu Y, Ge X, Zhao Z, Yao X. Production and characterization of intertribal somatic hybrids of *Raphanus sativus* and *Brassica rapa* with dye and medicinal plant *Isatis indigotica*. Plant Cell Reports. 2008;27(5):873–83. doi: 10.1007/s00299-008-0513-1 18264711 10.1007/s00299-008-0513-1

[25] He Q, Cai Z, Hu T, Liu H, Bao C, Mao W. Repetitive sequence analysis and karyotyping reveals centromere-associated DNA sequences in radish (*Raphanus sativus* L.). BMC plant biology. 2015;15(1):105. 25928652 10.1186/s12870-015-0480-y PMC4417506

[26] Li G, Quiros CF. Sequence-related amplified polymorphism (SRAP), a new marker system based on a simple PCR reaction: its application to mapping and gene tagging in Brassica. Theoretical and Applied Genetics. 2001;103(2):455–61.

[27] Doyle JJ, Doyle JL, Brown AHD. Analysis of a polyploid complex in glycine with chloroplast and nuclear DNA. Australian Systematic Botany. 1993;3(1):125–36.

[28] Vos P, Hogers R, Bleeker M, Reijans M, Lee TVD, Hornes M, et al AFLP: a new technique for DNA fingerprinting. Nucleic acids research. 1995;23(21):4407 7501463 10.1093/nar/23.21.4407 PMC307397

[29] Rohlf FJ. NTSYSpc, numerical taxonomy and multivariate analysis system. 1988;2 1.

[30] Chen J, Jing J, Zhan Z, Zhang T, Zhang C, Piao Z. Identification of novel QTLs for isolate-specific partial resistance to *Plasmodiophora brassicae* in *Brassica rapa*. Plos One. 2013;8(12):e85307 doi: 10.1371/journal.pone.0085307 24376876 10.1371/journal.pone.0085307 PMC3869933

[31] Rahman H. Review: Breeding spring canola (*Brassica napus* L.) by the use of exotic germplasm. Canadian Journal of Plant Science. 2013;93(3):363–73.

[32] Schelfhout CJ, Snowdon R, Cowling WA, Wroth JM. Tracing B-genome chromatin in *Brassica napus* × *B*. *juncea* interspecific progeny. Genome. 2006;49(11):1490–7. doi: 10.1139/g06-103 17426764 10.1139/g06-103

[33] Cronn R, Small RL, Haselkorn T, Wendel JF. Cryptic repeated genomic recombination during speciation in *Gossypium gossypioides*. Evolution. 2013;57(11):2475–89. 10.1111/j.0014-3820.2003.tb01493.x 14686525

[34] Jauhar PP, Peterson TS. Chromosome engineering of durum wheat with alien chromatin of diploid wheatgrass. Journal of Crop Improvement. 2009;23(4):319–31.

[35] Hagimori M, Nagaoka M, Kato N, Yoshikawa H. Production and characterization of somatic hybrids between the Japanese radish and cauliflower. Theoretical and Applied Genetics. 1992;84(7):819–24. 24201480 10.1007/BF00227390

[36] Pellan-Delourme R, Renard M. Identification of maintainer genes in *Brassica napus* L. for the male-sterility-inducing cytoplasm of *Diplotaxis muralis* L. Plant Breeding. 2006;99(2):89–97.

[37] Arumugam N, Mukhopadhyay A, Gupta V, Sodhi YS, Verma JK, Pental D, et al Synthesis of somatic hybrids (RCBB) by fusing heat-tolerant *Raphanus sativus* (RR) and *Brassica oleracea* (CC) with *Brassica nigra* (B). Plant Breeding. 2002;121(121):168–70.

[38] Heneen WK, Chen BY, Cheng BF, Jonsson A, Simonsen V, Jørgensen RB, et al Characterization of the A and C genomes of *Brassica Campestris* and *B*. *Alboglabra*. Hereditas. 2010;123(3):251–67.

[39] Mason AS, Nelson MN, Yan G, Cowling WA. Production of viable male unreduced gametes in Brassica interspecific hybrids is genotype specific and stimulated by cold temperatures. BMC plant biology. 2011;11(1):103. 21663695 10.1186/1471-2229-11-103 PMC3141635

[40] Jenczewski E, Eber F, Grimaud A, Huet S, Lucas MO, Monod H, et al *PrBn*, a major gene controlling homeologous pairing in oilseed rape (*Brassica napus*) haploids. 2003;164(2):645–53. 12807785 10.1093/genetics/164.2.645 PMC1462591

[41] Lee YP, Park S, Lim C, Kim H. Discovery of a novel cytoplasmic male-sterility and its restorer lines in radish (*Raphanus sativus* L.). Theoretical and Applied Genetics. 2008;117(6):905 doi: 10.1007/s00122-008-0830-3 18597066 10.1007/s00122-008-0830-3

[42] Hu X, Sullivan-Gilbert M, Kubik T, Danielson J, Hnatiuk N, Marchione W, et al Mapping of the Ogura fertility restorer gene *Rfo* and development of *Rfo* allele-specific markers in canola (*Brassica napus* L.). Molecular Breeding. 2008;22(4):663–74.

[43] Feng J, Primomo V, Li Z, Zhang Y, Jan CC, Tulsieram L, et al Physical localization and genetic mapping of the fertility restoration gene *Rfo* in canola (*Brassica napus* L.). Genome. 2009;52(4):401 doi: 10.1139/g09-016 19370095 10.1139/g09-016

[44] Li M, Qian W, Meng J, Li Z. Construction of novel *Brassica napus* genotypes through chromosomal substitution and elimination using interploid species hybridization. Chromosome Research. 2004;12(5):417–26. doi: 10.1023/B:CHRO.0000034722.66981.94 15252238 10.1023/B:CHRO.0000034722.66981.94

[45] Qi L, Friebe B, Peng Z, Gill BS. Homoeologous recombination, chromosome engineering and crop improvement. Chromosome Research. 2007;15(1):3–19. doi: 10.1007/s10577-006-1108-8 17295123 10.1007/s10577-006-1108-8

[46] Han H, Bai L, Su J, Zhang J, Song L, Gao A, et al Genetic rearrangements of six wheat-agropyron cristatum 6P addition lines revealed by molecular markers. Plos One. 2014;9(3):e91066 doi: 10.1371/journal.pone.0091066 24595330 10.1371/journal.pone.0091066 PMC3942500

[47] Cifuentes M, Garciaagüero V, Benavente E. A comparative analysis of chromosome pairing at metaphase I in interspecific hybrids between durum wheat (*Triticum turgidum* L.) and the most widespread Aegilops species. Cytogenetic & Genome Research. 2010;129(1–3):124. 20551603 10.1159/000313593

[48] Rieseberg LH, Willis JH. Plant speciation. Science. 2007;317(5840):910–4. doi: 10.1126/science.1137729 17702935 10.1126/science.1137729 PMC2442920

[49] Levin DA. The long wait for hybrid sterility in flowering plants. New Phytologist. 2012;196(196):666–70. 22966819 10.1111/j.1469-8137.2012.04309.x

