# Supplementary material for: Cytological and morphological analysis of hybrids between Brassicoraphanus, and Brassica napus for introgression of clubroot resistant trait into Brassica napus L
Source: PLoS One. 2017 May 15;12(5):e0177470. doi: 10.1371/journal.pone.0177470 (PMC5432170; doi:10.1371/journal.pone.0177470)
Supplement: S1 Table — Abnormal pollen fertility of F1 hybrids and BC1 individuals. (DOCX) [file pone.0177470.s001.docx]

**S1 Table. Pollen fertility in randomly selected BC1 individuals**

|  | **F_1_** | **BC_1_** | | | | | | | | | | | | | |
| --- | --- | --- | --- | --- | --- | --- | --- | --- | --- | --- | --- | --- | --- | --- | --- |
| **Plant material** | ARCC | 42-12 | 43-9 | 43-17 | 43-18 | 42-11 | 43-11 | 43-1 | 43-12 | 42-5 | 43-2 | 43-26 | 42-1 | 43-23 |  |
| **Mean±dev.st** | 4.86±2.06 | 19.45±3.60 | 6.44±1.88 | 5.64±2.32 | 33.23±4.03 | 16.69±1.71 | 33.36±2.68 | 26.99±2.98 | 7.42±3.84 | 9.68±3.72 | 18.70±1.21 | 30.42±6.17 | 17.91±4.35 | 20.98±9.22 |  |
