# Supplementary material for: Cytological and morphological analysis of hybrids between Brassicoraphanus, and Brassica napus for introgression of clubroot resistant trait into Brassica napus L
Source: PLoS One. 2017 May 15;12(5):e0177470. doi: 10.1371/journal.pone.0177470 (PMC5432170; doi:10.1371/journal.pone.0177470)
Supplement: S2 Table — AFLP analysis identified polymorphic bands among randomly selected 19 BC1 individuals. (DOCX) [file pone.0177470.s002.docx]

**S2 Table. Summary of AFLP bands in random selected BC_1_ individuals**

| **BC_1_** | **42-1** | **42-2** | **42-5** | **42-7** | **42-11** | **42-12** | **42-13** | **42-15** | **43-1** | **43-2** | **43-9** | **43-11** | **43-12** | **43-15** | **43-17** | **43-18** | **43-22** | **43-23** | **43-26** |
| --- | --- | --- | --- | --- | --- | --- | --- | --- | --- | --- | --- | --- | --- | --- | --- | --- | --- | --- | --- |
| Common bands | 86 | 88 | 89 | 86 | 88 | 88 | 87 | 85 | 90 | 86 | 89 | 89 | 87 | 88 | 90 | 89 | 87 | 87 | 88 |
| Unique bands in AACC | 71 | 81 | 70 | 70 | 75 | 71 | 74 | 78 | 70 | 73 | 69 | 73 | 71 | 72 | 73 | 72 | 71 | 71 | 81 |
| Unique bands in RRCC | 63 | 53 | 64 | 64 | 59 | 63 | 60 | 56 | 64 | 61 | 65 | 61 | 63 | 62 | 61 | 62 | 63 | 63 | 53 |
| New bands | 11 | 9 | 8 | 11 | 9 | 9 | 10 | 12 | 7 | 11 | 8 | 8 | 10 | 9 | 7 | 8 | 10 | 10 | 9 |
