# Supplementary material for: Cytological and morphological analysis of hybrids between Brassicoraphanus, and Brassica napus for introgression of clubroot resistant trait into Brassica napus L
Source: PLoS One. 2017 May 15;12(5):e0177470. doi: 10.1371/journal.pone.0177470 (PMC5432170; doi:10.1371/journal.pone.0177470)
Supplement: S3 Table — (DOCX) [file pone.0177470.s003.docx]

**S3 Table. Pollen fertility of backcross parent and resistant BC_2_ individuals**

|  |  | BC_2_ | | | | | | | | | |
| --- | --- | --- | --- | --- | --- | --- | --- | --- | --- | --- | --- |
| plant materials | HS 5 | 17-1 | 17-2 | 17-3 | 17-4 | 17-5 | 17-6 | 17-7 | 17-8 | 17-9 | 17-10 |
| Mean±dev.st | 100%±0 | 98.64±1.54 | 37.07±1.53 | 37.34±4.16 | 97.71±2.11 | 98.95±0.58 | 98.70±0.19 | 44.51±11.49 | 98.51±1.89 | 55.21±3.65 | 98.26±0.89 |
